# Growth and nitrogen fixation of legumes at increased salinity under field conditions: implications for the use of green manures in saline environments

**DOI:** 10.1093/aobpla/plv010

**Published:** 2015-02-06

**Authors:** Bas Bruning, Richard van Logtestijn, Rob Broekman, Arjen de Vos, Andrés Parra González, Jelte Rozema

**Affiliations:** 1 Systems Ecology, Department of Ecological Science, Faculty of Earth and Life Sciences, VU University Amsterdam, Amsterdam, The Netherlands; 2 Salt Farm Texel, Den Burg, The Netherlands; 3 Department of Biological Oceanography, Royal Netherlands Institute for Sea Research (NIOZ), Den Burg, The Netherlands

**Keywords:** Halophytes, *Melilotus officinalis*, salinity, *Sesbania*, symbiotic nitrogen fixation

## Abstract

The use of legumes as green manure can potentially increase crop productivity in saline environments and thus contribute to the sustainability of agricultural systems. Here, we present results from a field experiment conducted in the Netherlands that addressed the efficiency of nitrogen (N) fixation by a legume at varying salinities. We grew *Melilotus officinalis* in an agricultural field using drip irrigation with water salinity varying in electrical conductivity between 1.7 and 20 dS m^−1^. In the experiment, nearly 100 % of total plant N in *M. officinalis* was derived from symbiotic fixation at all but the highest salinity level (20 dS m^−1^). Our results indicated that this species derived substantial amounts of N via symbiotic fixation, the N becoming available in the soil (and thus available to crops) when cultivated legumes senesce and decompose. Based on the growth performance of *M. officinalis* and its ability to fix N at moderate soil salinity in our field experiments, we identified this species as a promising source for green manure in saline agriculture in temperate regions.

## Abstract (precis)

In this paper we identify yellow sweet clover (*Melilotus officinalis*) as a suitable candidate to be adopted as a green manure in saline agriculture. The plants perform well up to a third seawater salinity concentration and continue to get their nitrogen from symbiotic root bacteria. Alternatively, alfalfa (*Medicago sativa*) could be used since it shows similar tolerance to salinity. However, since the absolute biomass of yellow sweet clover is considerably higher, this plant would be our choice if the goal is to enrich soils with organic material and, specifically, with a sustainable input of nitrogen.

## Introduction

With a predicted global population of 9–10 billion people in 2050 (United Nations (UN), 2013) our ability to feed the world in the 21st century will depend upon our capacity to produce enough food. However, the impact of food production on the environment should be kept at a minimum. Even though global food production is not distributed equally over the human population, at present the quantity of food is sufficient for the world's population (Food and Agriculture Organization (FAO), 2002). This has been accomplished through a series of developments commonly referred to as the ‘green revolution’: increases in yields per hectare via higher-yielding crop varieties, optimizing the use of fertilizers and pesticides and improved irrigation practices. Though impressive, these developments are considered by many to be ultimately unsustainable and alternatives have to be found (Spiertz 2010; Coba de la Peña and Pueyo 2012). For example, global fertilizer production is responsible for ∼2 % of the world's energy consumption but largely makes use of non-renewable resources (Vance 2001; Foley *et al*. 2005).

One suggestion to expand our agricultural output with minimal impact on natural ecosystems is to use salt-affected lands for agricultural purposes. Large areas around the world are, through human activity or through natural causes, affected by high levels of soil salinity in ways that render such lands unsuitable for conventional agriculture because yields are unprofitable (Zahran 1999). Several options exist to raise yields to a viable level. First, naturally salt-tolerant species (halophytes) can be used as crops. Second, conventional crops can be improved through classical breeding so that they produce viable yields on salt-affected lands (Rozema and Flowers 2008; Munns *et al.* 2012; Rozema and Schat 2013). Third, techniques such as marker-assisted breeding or genetic engineering (reviewed by Dita *et al.* 2006, focussing on legumes) can be applied to generate salt-tolerant crop varieties. Any of these approaches would allow increased food production without necessitating conversion of more (natural) areas to agricultural fields, instead re-using those areas that have been abandoned because of increased salt levels.

Most agricultural systems depend on the application of nitrogen (N), as it is often the limiting nutrient (Vitousek and Howarth 1991). In conventional agricultural practices, N is applied as synthetic fertilizer, whereas in natural ecosystems—especially in arid and semi-arid regions—the main source of N is through biological N fixation by microorganisms (diazotrophs) (Cleveland *et al*. 1999; Zahran 1999). Legumes host N-fixing symbiotic bacteria (collectively called Rhizobia) that provide the host with N in return for photosynthates from the plant (Kneip *et al.* 2007). Through this process, legumes provide a net input of N in agricultural fields and 30–60 % of this added N (i.e. N derived from the atmosphere, Ndfa) is available for subsequent crops if (part of) the organic matter of the legume is incorporated into the soil (Chalk 1998). Ndfa can, at least partially, provide a viable solution for improving the sustainability of agriculture (Jensen and Hauggaard-Nielsen 2003).

To introduce a legume crop in saline agriculture requires either an effort to increase salt tolerance of currently used legumes or domestication of a naturally salt-tolerant legume. This paper will explore the latter option: whether symbiotic N fixation is possible at high soil salinities. Nitrogen fixation in root nodules in higher plants is often stated to be more sensitive to salinity than plant growth (Djekoun and Planchon 1991) and all stages in nodule formation and nodule functioning are negatively affected by salinity (Coba de la Peña and Pueyo 2012; Bruning and Rozema 2013). If legumes are to be adopted as green manures in salt-affected areas, it is essential that their N-fixing capabilities are not disproportionally affected by salinity. It is therefore very important to assess the effects of salinity on the efficiency of symbiotic N fixation in suitable legume species under field conditions.

While research has been carried out on salinity tolerance in legumes, much of the research focussed on commercially important or model species such as *Glycine max* and *Medicago truncatula*. For the research reported here, we focussed on a relatively salt-tolerant species, *Melilotus officinalis*. We also grew *Medicago sativa* on the same plots, some results of which are included in the **Supporting Information**. The genus *Melilotus* is one of the most salt tolerant and waterlogging tolerant of temperate legumes (Rogers *et al.* 2008). *Medicago sativa* is also considered relatively salt tolerant (moderately sensitive according to Munns and Tester 2008) and still grows at 250 mM NaCl (Noble *et al.* 1984). Using this species, we assessed the effect of various salinities on N-fixing efficiency with the aim of identifying a species suitable as green manure in saline agriculture. Specifically, we asked: what is the effect of increasing soil salinity on the efficiency of N fixation in *M. officinalis*?

## Methods

### Research location and irrigation strategy

Field experiments were performed with the legume species *Medicago sativa* and *M. officinalis* on an experimental field on Texel (53.012837°N, 4.755306°E), The Netherlands in 2013 and in 2014. In this article we will report only the results of the field trial of 2014. The experimental area consisted of a field (40 × 224 m) divided into 56 plots (8 × 20 m) with seven salt concentrations each replicated eight times and randomly distributed over the area. In the year prior to the experiment, the soil, which was mainly sand (3 % clay, 2 % organic matter), was homogenized by a large power shovel mixing the top 1 m of soil for 3 days. The plots were drip irrigated daily with 12 mm m^−2^ day^−1^ so that the soil moisture never dropped <80 % of the soil water-holding capacity. Individual drip lines were 40 cm apart, with drippers at 30 cm intervals. The irrigation water was a mixture of fresh water from a nearby rainwater basin and natural salt water from a nearby ditch fed from the Waddensea, with a conductivity of around 35 dS m^−1^. Fresh and saline waters were mixed using a custom-built proportional-integral-derivative (PID) controller with frequency-regulated pumps from both water sources, which allowed time-based automatic pulse irrigation. Average salinity levels of the irrigation water during an irrigation event reached a minimum of 0.5 dS m^−1^ accuracy compared with the target level. Drainage pipes located 60 cm below the surface, with 5 m spacing between any two pipes aided rapid drainage of the daily irrigation water and aeration of the soil.

Target electrical conductivities of the drip irrigation water were: 2, 4, 8, 12, 16 and 20 dS m^−1^.

### Soil salinity

Soil salinity was monitored during the experiment by means of soil samples (collected in all plots, three times during the experiment) and by sampling pore water (measured every 2 weeks). Soil samples in each plot were produced by mixing three individual samples from the top 30 cm of soil and dried at 70 °C for 48 h. Soil salinity was estimated in solutions derived from 1 : 2 mixtures (v : v, soil : water) and saturated pastes (29 samples). There was a strong correlation between the results from the 1 : 2 method (EC_1 : 2_) and the saturated paste method (EC_e_), with EC_e_ = EC_1 : 2_ × 5.33 (*r*^2^ = 0.94). All soil samples in this paper are expressed as EC_e_. Soil pore water was collected by means of suction cups that were placed at three different depths (0–10, 20–30 and 50–60 depth) in half of the plots (four of the eight replicates per salinity). Since no large variation occurred between the electrical conductivity of the soil pore water (EC_pw_) at the different depths, the average of the three depths was taken. There was a strong correlation between EC_pw_ and EC_e_ (EC_e_ = 0.69 × EC_pw_, with *r*^2^ = 0.82), and values of EC_pw_ are expressed as EC_e_ in this paper.

### Plant material

The experiment involved the legume species *M. officinalis*, seeds of which were obtained from B & T World Seeds, France (http://b-and-t-world-seeds.com/). Since *Medicago sativa* was grazed just before harvest we did not include data on this species in the main text of this article [**see** **Supporting Information**]. Per plot of 8 × 20 m, a 1.25 × 1.25 m subplot was used for each species. Seeds were sown in four rows on March in 2013 and in April in 2014 and grown for 4 months in each year. Plants were grown on the same 8 × 20 m plots but on different sub-plots, for different experiments. The data for the 2 years were very similar so here we present data from 2014.

At the start of the season, a bag made of water permeable cloth (https://www.google.nl/search?q=worteldoek&client=firefox-a&hs=Rxg&rls=org.mozilla:nl:official&channel=sb&biw=1680&bih=913&source=lnms&tbm=isch&sa=X&ei=XRFrVOaEKMy8PYDjgLAJ&ved=0CAYQ_AUoAQ; 20 × 20 × 50 cm) was dug in the soil into which the seeds were sown. The bag was designed to provide, by the end of the season, a better harvest of all the roots and nodules of a plant. The bags, which were kept completely free of other plants, were dug out at harvest. Roots and shoots were carefully washed with demineralized water. Then roots, shoots and nodules were separated (nodules picked off by hand) and dried in an oven at 70 °C for 48 h and dry weights were determined.

The dried shoots were ground with a ball mill, and 3–4 mg of the material used to estimate N content and N isotopic composition using an elemental analyser (NC2500, ThermoQuest Italia, Rodano, Italy) coupled online to a stable isotope ratio mass spectrometer (Delta^plus^; Thermofinnigan, Bremen, Germany). Two non-legume species (*Chenopodium album* and *Brassica napus*) were harvested as reference plants for the calculations of the percentage symbiotically N fixed (Ndfa) by the legumes (Shearer and Kohl 1986). The percentage of Ndfa was calculated as follows:

$$\text{Ndfa}=\frac{\delta {}^{15}\text{N}\;\text{reference plant}\;-\delta {}^{15}\text{N}\;\text{legume}}{\delta {}^{15}\text{N}\;\text{reference plant}\;}$$

### Statistics

We performed two-way ANOVAs with salinity and species as independent variables and Ndfa, N content and ^13^C values as dependent variables. Tukey's post-hoc test was used to determine significant differences between salt levels. All data were analysed using SPSS 21 statistical software.

## Results

### Field experiment

#### Soil parameters

**Figure 1.**
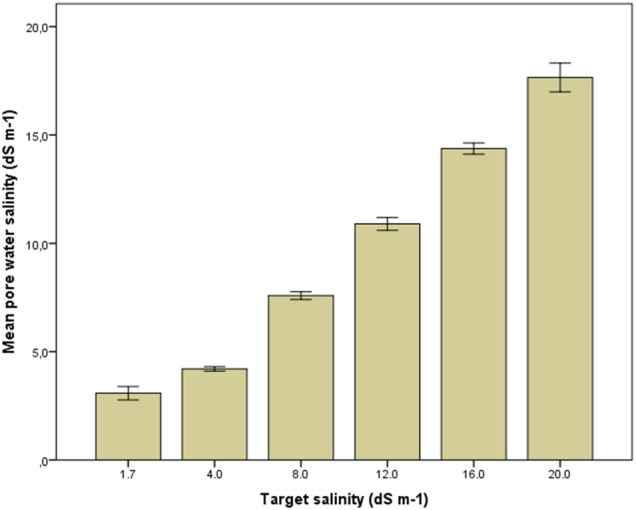
Electrical conductivity of the pore water in 2014. Data based on measurements using various methods (see Methods section) and are all expressed as EC_e_ based on correlations between the three methods used. Bars indicate average values with standard error of the mean.

The soil salinity closely followed the salinity of the drip irrigation water (Fig. 1). The irrigation water itself was measured regularly and never deviated from the target salinity by more than 0.1 dS m^−1^ (data not shown). Carbon and N contents of the soil were similar in all plots (data from 2013, no data for 2014). The results of a soil analysis are shown in Table 1, of the 2013 season.

**Table 1.** Soil C/N ratios and total nitrogen of the experimental field in 2013 (no data available for 2014). Data are averages of duplicate measurements in two different plots for each salt treatment.

	Salinity					
	1	4	8	12	16	20
C/N ratio	6	6	6	7	6.5	5.5
Total nitrogen (mg kg^−1^)	1810	1820	1585	1630	1630	1410

### Plant growth

**Figure 2.**
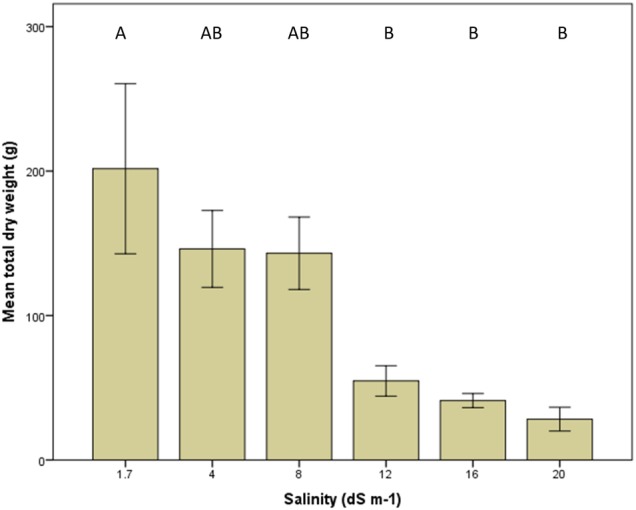
Total plant dry weight (roots plus shoots) from *M. officinalis* in 2014. Different letters above the bars reflect significant differences between the treatments [ANOVA, *F*_5, 37_ = 6.0, *P* = 0.001 (post-hoc Tukey's HSD multiple comparison test, *P* ≤ 0.05)]. Bars indicate average values with standard error of the mean.

Plant growth of *M. officinalis* was negatively affected by salinity. Figure 2 shows biomass data at the varying salinity levels. After 8 dS m^−1^, plant biomass was significantly lower than at lower salinities, but there was no significant difference between plant biomass at 12, 16 and 20 dS m^−1^. Hares ate many of the *Medicago sativa* plants, rendering biomass measurements uninformative and hence the data are not shown.

### Symbiotic N fixation

δ^15^N values of the four reference plant species did not differ significantly in both years. A mean value of 9.58‰ was used for the calculations of Ndfa.

**Figure 3.**
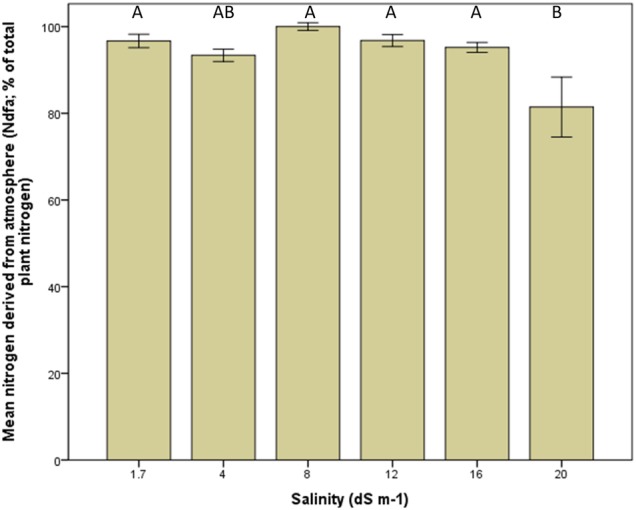
Nitrogen derived from the atmosphere (Ndfa) as a percentage of total plant N in *M. officinalis* at various irrigation salinities in 2014. Different letters above the bars reflect significant differences between the treatments [ANOVA, *F*_5, 39_ = 4.8, *P* = 0.002 (post-hoc Tukey's HSD multiple comparison test, *P* ≤ 0.05)]. Bars indicate average values with standard error of the mean.

The Ndfa was negatively affected by salinity but only at the highest treatment (20 dS m^−1^) (Fig. 3; ANOVA: *F*_5, 39_ = 4.8, *P* = 0.002, post-hoc Tukey's HSD multiple comparison test, *P* ≤ 0.05). Close to 100 % of the N was derived from the atmosphere at all but the highest salinity level, dropping to around 80 % at 20 dS m^−1^. In *Medicago sativa*, around 90 % of total plant N was derived from the atmosphere, dropping to 70 % at 16 dS m^−1^ and to 43 % at 20 dS m^−1^ **[see Supporting Information**].

### Total plant N

**Figure 4.**
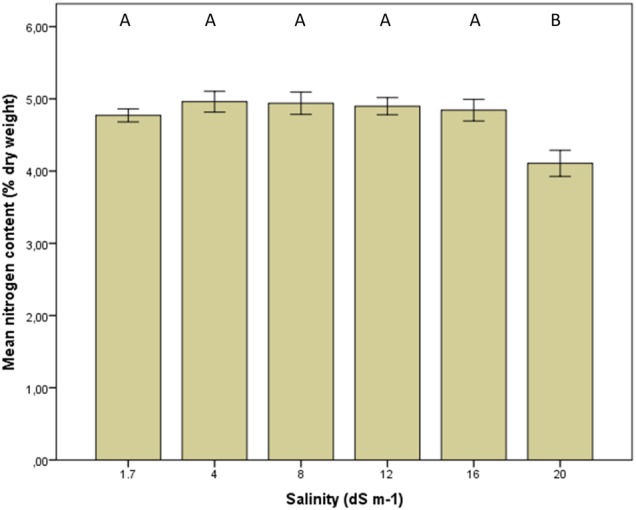
Total plant nitrogen in *M. officinalis* at increasing salinity. Different letters above the bars reflect significant differences between the treatments [ANOVA, *F*_5, 39_ = 4.7, *P* = 0.002 (post-hoc Tukey's HSD multiple comparison test, *P* ≤ 0.05)]. Bars indicate average values with standard error of the mean.

Total plant N was affected little by salinity even though there was still a significant effect of treatment (for *M. officinalis*: ANOVA, *F*_5, 39_ = 4.7, *P* = 0.002, post-hoc Tukey's HSD multiple comparison test, *P* ≤ 0.05, Fig. 4). At all but the highest salinities total plant N was close to 5 %, dropping to ∼4 % at 20 dS m^−1^. Data on total plant N in *Medicago sativa* is given in **Supporting Information**. The mean N content was ∼3.5 % at salinities up to 16 dS m^−1^, dropping to 3 % at 16 dS m^−1^ and to 2.7 % at 20 dS m^−1^.

## Discussion

We present data aimed at answering the question: is symbiotic N fixation reduced at high soil salinities? Our findings indicated that this was indeed the case. Additionally, as stated in the methods we performed this experiment in two consecutive years and the results of both years are nearly identical. These findings strongly suggest that the capacity of symbiotic N fixation would not limit the adoption of legumes as green manure in a saline agricultural system, but rather that the general salt tolerance of the legumes (growth performance) would be a limiting factor. Both species used in this study showed good salt tolerance and could be serious candidates for green manure in fields with salinities between 1.7 and 12 or even 16 dS m^−1^.

The soil salinity [based on soil pore water, saturated pastes and the 1 : 2 (v : v) method (see Methods section)] closely followed the salinity of the drip irrigation. This was the case in both years we performed the experiments (data not shown).

Both species maintained their N content upon increasing salinity, with a reduction being recorded only when the salinity of the irrigation water reached 20 dS m^−1^ for *M. officinalis*. The value of a legume as a green manure is a combination of its N content, total biomass and Ndfa. Our results suggest that the effectivity of *M. officinalis* as a green manure under saline conditions is not negatively affected through its N content.

How do our findings compare with other published results? Few data are available on N fixation at salinities of 250 mM NaCl (∼25 dS m^−1^) and above. Bruning and Rozema (2013) compared the relative performance of growth and N fixation of 16 legumes from published studies, where the study with the highest salinity was 120 mM NaCl (around 12 dS m^−1^) and then for only 2 of the 16 species (*Acacia nilotica* and *Prosopis juliflora*). The remaining studies were done at much lower salinities, reflecting a limited salt tolerance for those species under study. As a phylum, the *Fabaceae* are slightly below average in salt tolerance, expressed as the number of salt-tolerant species in the eHALOPH database divided by the total number of species in the phylum (0.22 % of species in the clade are salt tolerant; average of all Angiosperms: 0.49 %: Bruning *et al.* in preparation). The eHALOPH database does not use as strict a definition for halophytism as Flowers and Colmer (2008), who consider a plant a halophyte when it is able to complete its life cycle at 200 mM NaCl or more. The eHALOPH database contains species that are considered salt tolerant when they are able to perform well at a minimum of 7–8 dS m^−1^, so around 80 mM of NaCl. Despite these different definitions, it is likely that true halophytism as in the definition by Flowers and Colmer (2008) must also exist in the *Fabaceae* (i.e. this paper).

We searched the literature for data on growth and N fixation at high salinity (higher than 200 mM NaCl) levels in symbiotically N-fixing species. Table 2 summarizes data extracted from four publications on N fixation and growth of four different N-fixing species. The different publications vary greatly in their methods concerning growth conditions (soil or hydroponics) and duration, from just 25 days to almost 6 months, but all species have been tested up to high (>150 mM of NaCl) salinity. For ease of comparison, despite differences in methodology and growing times, we expressed both biomass production and N fixation relative to the species' performance at 0 mM NaCl.

**Table 2.** Data on symbiotic nitrogen fixation from the scientific literature. Nitrogen fixation performance at different salinities is calculated on the basis of the Acetylene Reduction Assay (ARA at 0 mM NaCl taken as 100 %) for all species except for *Sesbania sesban*, in which case relative nodule dry weight is taken as a proxy for N fixation. *For *Sesbania sesban*: reduction in nodule dry weight as compared to nodule dry weight at 0 mM; for all other species reduction in ARA as compared to ARA at 0 mM NaCl. **Fresh weight for *S. rostra*, the other species total dry weight. For all species, weight is expressed relative to the weight at 0 mM of NaCl. ***Actinorrhizal species (not a legume). ^1^Mahmood *et al*. (2008), ^2^Hopmans *et al*. (1983), ^3^Jungklang (2003), ^4^Ng (1987).

Species	NaCl (mM)	Nitrogen content (%)	Relative N-fixation performance*	Relative weight**	Duration of treatment (days)	Method
*Sesbania sesban*^1^	0	0.48	100	100	80	3 L soil in pots
	34	0.38	79	91.2	80	3 L soil in pots
	69	0.35	73	82.1	80	3 L soil in pots
	103		71	76.9	80	3 L soil in pots
	131	0.33	64	72.8	80	3 L soil in pots
	172	0.04	31	69.9	80	3 L soil in pots
*Acacia dealbata*^2^	0	1.79	100	100	25	200 g sandy loam in pots
	25	1.89	99	93.3	25	201 g sandy loam in pots
	75	1.62	71	88.4	25	202 g sandy loam in pots
	150	1.55	46	86.3	25	203 g sandy loam in pots
	250	1.67	17	58.9	25	204 g sandy loam in pots
*Sesbania rostrata*^3^	0	3.92	100	100	50	Hydroponics
	25	3.9	86	90	50	Hydroponics
	50	3.73	113	83.3	50	Hydroponics
	100	3.6	84	71.1	50	Hydroponics
	150	3.32	5	41.1	50	Hydroponics
	200	3.62	0	27.8	50	Hydroponics
*Casuarina equisetifolia*^4^^,^***	0	1.9	100	100	168	Sand in pots
	20	1.9	108	81.9	168	Sand in pots
	50	1.5	83	136.6	168	Sand in pots
	100	1.6	85	132.1	168	Sand in pots
	200	1.8	98	104.2	168	Sand in pots
	500	1.7	42.5	41.9	168	Sand in pots

One of the four species (*Casuarina equisetifolia*) is an actinorhizal species, i.e. not a legume. This species is the most salt tolerant for which we could find N-fixing data: growth and N fixation are not affected at 200 mM NaCl. The two *Sesbania* species appear to be the least salt tolerant of the listed species. Our data suggest that *M. officinalis* is quite as tolerant as any of the species listed in Table 2 and fixes N at the salt concentration used by Flowers and Colmer (2008) to define a halophyte, although the little data that we could find, in the literature and our studies on Texel, appear to show that it is rare for N fixation to happen above 200 mM NaCl. Unfortunately, however, such a limited number of species precludes any formal (meta)analysis.

There are costs associated with symbiotic N fixation in terms of units carbon invested per unit N obtained (Phillips 1980), because of the ATP requirements of nitrogenase and because the host plant supplies the symbiotic Rhizobia with carbon compounds. This energy requirement of legumes that depend on symbiotic N fixation is likely the reason for their sensitivity compared with growing on mineral N (i.e. supplied with ammonium ions) (Delgado *et al.* 1994). Similarly, the increased stress on a plant associated with increasing salinity in the soil and the concomitant increased energy expenditure is probably the reason why some authors have found symbiotic N fixation to be more sensitive to salinity than general growth of the host plant (Delgado *et al.* 1994). Exposure to salinity requires plants to respond in several ways, such as the synthesis of compatible solutes and sodium sequestration in the vacuole, which also must pose energetic constraints to plant performance. However, in Bruning and Rozema (2013), there were no apparent differences in the slopes of N fixation and growth at increasing salinity. The interaction between salinity and symbiotic N fixation deserves further attention.

## Conclusions

Based on the definition of halophytes by Flowers and Colmer (2008), halophytic N fixation does exist. Here we have identified from our experiments on Texel, one species, *M. officinalis*, that is able to grow and fix N symbiotically at high soil salinity levels (20 dS m^−1^) and from the literature three species that fix N and four that will grow at at least 200 mM of NaCl. Hence, halophytic N fixation does occur but does not seem to be widespread in the large plant family of the Fabaceae. The percentage of plant N fixed in both species grown on Texel is very high, being close to 100 %. However, plants were harvested near the end of the completion of the life cycle and this is the moment when Ndfa is known to be highest and legumes receiving their total plant N from symbiotic fixation have been previously reported (Bergersen 1982). Additionally, the sandy soil on the test location is poor in nutrients and our plots were not fertilized for these experiments. Nevertheless, our results show that symbiotic N fixation persists under relatively high salt concentrations (at least one-quarter sea water strength) and this suggests legumes can serve as a green manure in a saline agricultural system. This improves the sustainable character already associated with saline agriculture.

## Sources of Funding

This research was funded by the Waddenfonds, project Zilt Perspectief.

## Contributions by the Authors

B.B. performed the field experiment, analysed the data and wrote most of the manuscript; R.v.L. performed the ^15^N isotope analyses, helped with further processing of the samples and helped write the manuscript; R.B. assisted with field work and processing of the plant material; A.de V. assisted with the fieldwork and helped write the manuscript; A.P.G. helped with the fieldwork and processing of the plant material and J.R. helped write the manuscript.

## Conflict of Interest Statement

None declared.

## Supporting Information

The following additional information is available in the online version of this article –

**Table S1.** Mean and standard deviations of total plant nitrogen and nitrogen derived from atmosphere of *Medicago sativa* in 2014.

## References

[PLV010C1] Bergersen FJ 1982 Root nodules of legumes: structure and functions. Chichester, NY: Research Study Press.

[PLV010C2] Bruning B Rozema J 2013 Symbiotic nitrogen fixation in legumes: perspectives for saline agriculture. Environmental and Experimental Botany 92:134–143. 10.1016/j.envexpbot.2012.09.001

[PLV010C3] Chalk PM 1998 Dynamics of biologically fixed N in legume-cereal rotations: a review. Australian Journal of Agricultural Research 49:303–316. 10.1071/A97013

[PLV010C4] Cleveland CC Townsend AR Schimel DS Fisher H Howarth RW Hedin LO Perakis SS Latty EF Von Fischer JC Elseroad A Wasson MF 1999 Global patterns of terrestrial biological nitrogen (N_2_) fixation in natural ecosystems. Global Biogeochemical Cycles 13:623–645. 10.1029/1999GB900014

[PLV010C5] Coba de la Peña T Pueyo JJ 2012 Legumes in the reclamation of marginal soils, from cultivar and inoculant selection to transgenic approaches. Agronomy for Sustainable Development 32:65–91. 10.1007/s13593-011-0024-2

[PLV010C6] Delgado MJ Ligero F Lluch C 1994 Effects of salt stress on growth and nitrogen fixation by pea, faba-bean, common bean and soybean plants. Soil Biology and Biochemistry 26:371–376. 10.1016/0038-0717(94)90286-0

[PLV010C8] Dita MA Rispail N Prats E Rubiales D Singh KB 2006 Biotechnology approaches to overcome biotic and abiotic stress constraints in legumes. Euphytica 147:1–24. 10.1007/s10681-006-6156-9

[PLV010C9] Djekoun A Planchon C 1991 Water status effect on dinitrogen fixation and photosynthesis in Soybean. Agronomy Journal 83:316–322. 10.2134/agronj1991.00021962008300020011x

[PLV010C11] Flowers TJ Colmer TD 2008 Salinity tolerance in halophytes. New Phytologist 179:945–963. 10.1111/j.1469-8137.2008.02531.x 18565144

[PLV010C12] Foley JA DeFries R Asner GP Barford C Bonan G Carpenter SR Chaplin FS Coe MT Daily GC Gibbs HK Helkowski JH Holloway T Howard EA Kucharik CJ Monfreda C Patz JA Prentice IC Ramankutty N Snyder PK 2005 Global consequences of land use. Science 309:570–574. 10.1126/science.1111772 16040698

[PLV010C13] Food and Agriculture Organization, International Fund for Agricultural Development, World Food Program. 2002 Reducing poverty and hunger, the critical role of financing for food, agriculture, and rural development. http://www.fao.org/docrep/005/y7352e/y7352e00.htm.

[PLV010C15] Hopmans P Douglas LA Chalk PM 1983 Nitrogen fixation associated with *Acacia dealbata* link. Seedlings as estimated by the acetylene reduction assay. Australian Journal of Botany 31:331–339. 10.1071/BT9830331

[PLV010C16] Jensen ES Hauggaard-Nielsen H 2003 How can increased use of biological N_2_ fixation in agriculture benefit the environment? Plant and Soil 252:177–186. 10.1023/A:1024189029226

[PLV010C37] Jungklang J 2003 Physiological and biochemical mechanisms of salt tolerance in Sesbania rostrata Brem. & Oberm. PhD Thesis, Division of Applied Biochemistry, University of Tsukuba, Ibaraki, Japan.

[PLV010C17] Kneip C Lockhart P Voβ C Maier U-G 2007 Nitrogen fixation in eukaryotes – new models for symbiosis. BMC Evolutionary Biology 7:55–66. 10.1186/1471-2148-7-55 17408485 PMC1853082

[PLV010C19] Mahmood A Athar M Qadri R Mahmood N 2008 Effect of NaCl salinity on growth, nodulation and total nitrogen content in *Sesbania sesban*. Agriculturae Conspectus Scientificus 73:137–141.

[PLV010C21] Munns R Tester M 2008 Mechanisms of salinity tolerance. Annual Review of Plant Biology 59:651–681. 10.1146/annurev.arplant.59.032607.092911 18444910

[PLV010C22] Munns R James RA Xu B Athman A Conn SJ Jordans C Byrt CS Hare RA Tyerman SD Tester M Plett D Gilliham M 2012 Wheat grain yield on saline soils is improved by an ancestral Na^+^ transporter gene. Nature Biotechnology 30:360–364. 10.1038/nbt.2120 22407351

[PLV010C23] Ng BH 1987 The effects of salinity on growth, nodulation and nitrogen fixation of *Casuarina equisetifolia**.* Plant and Soil 103:123–125. 10.1007/BF02370676

[PLV010C24] Noble CL Halloran GM West DW 1984 Identification and selection for salt tolerance in lucerne (*Medicago sativa* L.). Australian Journal of Agricultural Research 35:239–252.

[PLV010C26] Phillips DA 1980 Efficiency of symbiotic nitrogen fixation in legume*s.* Annual Review of Plant Physiology 31:29–49. 10.1146/annurev.pp.31.060180.000333

[PLV010C27] Rogers ME Colmer TD Frost K Henry D Cornwall D Hulm E Deretic J Hughes SR Craig AD 2008 Diversity in the genus Melilotus for tolerance to salinity and waterlogging*.* Plant and Soil 304:89–101. 10.1007/s11104-007-9523-y

[PLV010C28] Rozema J Flowers TJ 2008 Crops for a salinized world. Science 322:1478–1480. 10.1126/science.1168572 19056965

[PLV010C29] Rozema J Schat H 2013 Salt tolerance of halophytes, research questions reviewed in the perspective of saline agriculture. Environmental and Experimental Botany 92:83–95. 10.1016/j.envexpbot.2012.08.004

[PLV010C30] Shearer G Kohl DH 1986 N_2_-fixation in field settings: estimations based on natural 15N abundance*.* Australian Journal of Plant Physiology 13:699–756.

[PLV010C32] Spiertz JHJ 2010 Nitrogen, sustainable agriculture and food security. A review. Agronomy for Sustainable Development 30:43–55. 10.1051/agro:2008064

[PLV010C33] United Nations, Department of Economic and Social Affairs, Population Division. 2013 World population prospects: the 2012 revision, key findings and advance tables. Working Paper No. ESA/P/WP.227. http://esa.un.org/wpp/documentation/publications.htm.

[PLV010C34] Vance CP 2001 Symbiotic nitrogen fixation and phosphorus acquisition. Plant nutrition in a world of declining renewable resources*.* Plant Physiology 127:390–397. 10.1104/pp.010331 11598215 PMC1540145

[PLV010C35] Vitousek PM Howarth RW 1991 Nitrogen limitation on land and in the sea: how can it occur? Biogeochemistry 13:87–115. 10.1007/BF00002772

[PLV010C36] Zahran HH 1999 Rhizobium-legume symbiosis and nitrogen fixation under severe conditions and in an arid climate. Microbiology and Molecular Biology Review 63:968–989. 10.1128/mmbr.63.4.968-989.1999 PMC98982 10585971

